# Blockchain technology for secure and efficient nephrology data management

**DOI:** 10.1093/ckj/sfag201

**Published:** 2026-06-12

**Authors:** David R Evans, Ömer Tarik Özyilmaz, Huifang Xue, Michele Provenzano, Ning Qu, Stephan J L Bakker, Martin H de Borst, Uwe J F Tietge, Tamas Szili-Torok

**Affiliations:** Department of Internal Medicine, Division Nephrology, University Medical Center Groningen, University of Groningen, Groningen, The Netherlands; Department of Internal Medicine, Division Nephrology, University Medical Center Groningen, University of Groningen, Groningen, The Netherlands; Bernoulli Institute for Mathematics, Computer Science, and Artificial Intelligence, University of Groningen, Groningen, The Netherlands; Department of Internal Medicine, Division Nephrology, University Medical Center Groningen, University of Groningen, Groningen, The Netherlands; Nephrology, Dialysis and Transplant Unit, “SS. Annunziata” Hospital, Cosenza, Italy; Tianjin University Medical School, Tianjin, China; Graduate School of Medical Sciences, University Medical Center Groningen, University of Groningen, Groningen, The Netherlands; Department of Internal Medicine, Division Nephrology, University Medical Center Groningen, University of Groningen, Groningen, The Netherlands; Department of Internal Medicine, Division Nephrology, University Medical Center Groningen, University of Groningen, Groningen, The Netherlands; Division of Clinical Chemistry, Department of Laboratory Medicine, Karolinska Institutet, Stockholm, Sweden; Clinical Chemistry, Karolinska University Laboratory, Karolinska University Hospital, Stockholm, Sweden; Department of Internal Medicine, Division Nephrology, University Medical Center Groningen, University of Groningen, Groningen, The Netherlands

**Keywords:** big data, Blockchain, data management, data science, smart contracts

## Abstract

Rapid advancements in digital healthcare technologies have created new opportunities in nephrology, but they have also increased the fragmentation of datasets, complexity of data sharing, data privacy and security risks, and a lack of data verifiability and reliability (provenance). While several technological solutions have been developed to address these concerns individually, blockchain has been suggested as a unified solution for secure and efficient data management through several features. These include immutability, interoperability, and programmability, which provide a powerful framework that extends the utility of blockchain beyond data governance in nephrology. Blockchain also has potential applications in pharmaceutical and biobanking quality assurance and in the reliability of medical devices through auditable maintenance logs, making it a promising candidate to address several challenges in patient care and nephrology research. Despite this promise, practical challenges arise between the features of blockchain, such as immutability, and legislation, including the General Data Protection Regulation (GDPR). Furthermore, the value of real-world adoption in nephrology remains uncertain due to scalability, computational overheads, and implementation costs that may outweigh its advantages in many settings.

## INTRODUCTION

Nephrology is a complex field composed of diverse subspecialties usually distributed across different physical locations. For example, chronic kidney disease (CKD), hypertension and diabetes may be treated in primary, secondary and tertiary hospitals while dialysis and transplantation patients may be treated in specialized centers [1]. For efficient patient care and research that aims to capture the complex pathophysiology of CKD development, robust data harmonization across these facilities is essential. Modern nephrology frequently integrates clinical, laboratory and imaging data with continuous information from point-of-care devices (e.g. from glucose monitors) [2], exhibiting the volume, velocity and heterogeneity of ‘Big Data’ [3]. Big data represents an unprecedented opportunity for the development of more accurate predictive models for personalized medicine, provided that effective data management is in place. In practice, however, heterogeneous clinical data is often not integrated into one detailed clinical picture. Instead, a large proportion of data, generated and stored across diverse domains, remains isolated in what are known as ‘data silos’, critically limiting their clinical and research value. This problem naturally occurs as a consequence of inter-institutional incompatibility in data management but has also been reported to occur within individual institutions. Data fragmentation of this nature has been linked with a higher risk of adverse events such as increased frequency of emergency hospitalizations, greater use of redundant diagnostic tests, and increased healthcare costs in patients with chronic illness [4]. Conventional approaches to addressing data interoperability primarily focus on standardizing data formats and enabling exchange via application programming interfaces (APIs), which can effectively address the mechanics of data transfer, but have not eliminated data siloing. However, barriers related to governance, verifiable trust, data provenance, and integrity may represent more fundamental limitations to meaningful interoperability than data exchange itself.

Several technological advancements have been developed to address these challenges. Among these, blockchain technology has attracted significant attention over the last decade due to its potential to support secure data exchange, decentralized governance, auditable data provenance, and data integrity across distributed healthcare systems, while also enabling automated management of data through smart contracts [5]. Blockchain refers to a distributed ledger technology designed to record transactions in blocks of a linear chain for improved traceability and tamper-resistance. However, an apparent mismatch between attention directed toward blockchain and its adoption in real-world medical data management is noteworthy. The published literature on blockchain in healthcare is dominated by proof-of-concept studies and pilot applications, particularly in adjacent areas of healthcare such as medical supply chain management, while nephrology-specific publications remain especially sparse.

Importantly, blockchain should be compared with current best practice: conventional databases combined with access controls, standardized data formats, and application programming interfaces may provide comparable functionality with lower complexity and cost. This review provides a technical background of blockchain and critically examines its feasibility, potential benefits, and limitations for secure and efficient data management in nephrology.

### The principles of blockchain technology

**Figure 1:**
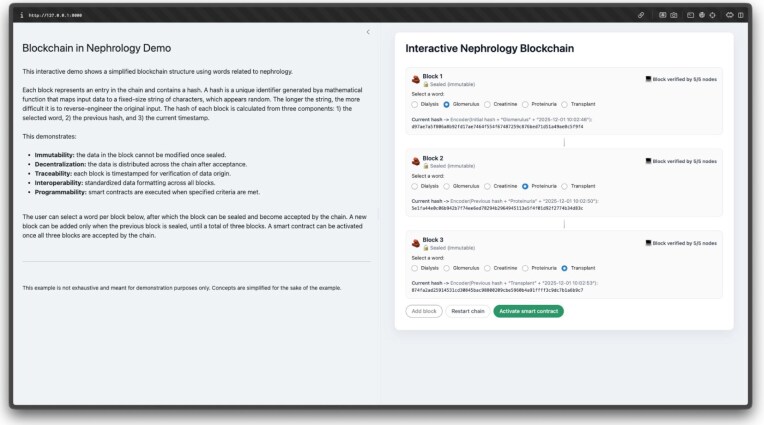
Key principles of blockchain technology, shown by the interactive web application developed.

A blockchain is a time-stamped, immutable (*defined as unable to be changed once its written*) archive of data organized as a sequential and connected, or chained, series of blocks [6, 7]. Each block in the chain consists of at least three components, the data that the block represents, such as a word, a sentence or a monetary transaction, a unique hash of that data, and the unique hash of the previous block [7, 8]. The hash is a sequence of numbers and letters, which can be derived by cryptographic functions, such as by the SHA256 function [9]. In essence, the moment a block of data is added to the chain, the previous unique hash is used to identify its current location on the chain, and the new unique hash is used to ensure the validity of the data. In the case that anyone maliciously or accidently tampers with, or changes the data before the last block in the chain, the current hash will be updated, rendering the subsequent blocks invalid. This ensures the first principle of blockchains: immutability. This means that data or blocks on the chain can only be replaced when all subsequent blocks are also regenerated to use the updated hash of the changed block. For example, given a series of blocks A, B, and C, a change in block B will trigger a change in the hash of block B and block C (since it relies on the hash from block B). The second principle of blockchains is decentralization. This means that instead of being managed on a single centralized computer, the entire blockchain is distributed as copies to each node (or computer) in the network. To this end, a majority stakeholder consensus must be reached before any new additions or modifications can be made to the chain, where a ‘majority’ can be defined per the blockchain rules (e.g. >50%, >75%, or including veto powers). This facilitates transparency and data provenance (*defined as traceability to the source data*), while reducing the risk of data tampering. Figure 1 shows an interactive web application which illustrates the key principles of blockchain technology (accessible from *tamas875.github.io/web-applications*).

**Figure 2:**
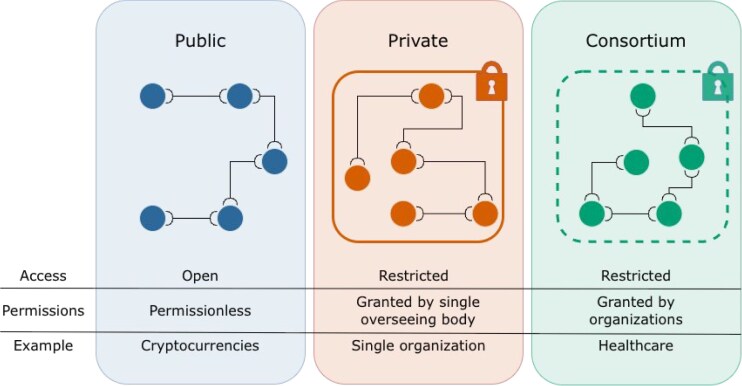
Summary of the different types of blockchains.

Three types of blockchain are currently used (Fig. 2) [10, 11]. The most commonly used version is the *public blockchain*, where anyone can access and participate in the chain, as in the case of cryptocurrencies. *Private blockchains* have permissioned access, meaning that only participants authorized by a centralized body are allowed access to the chain [12, 13]. The third type is the *consortium* or *federated blockchain*, a subtype of private blockchain used between organizations and governed through permissions. In this case, some organizations have more access than others, yet no single organization acts as the governing body of the chain. The consortium blockchain lends itself well to multi-institution collaborations common in nephrology research and clinical practice, as we will discuss in the next section [14, 15].

### Data management in modern nephrology: challenges and potential solutions

#### Decentralization & interoperability

To ensure that the data collected in clinical practice and used in clinical research is valid and trustworthy, it needs to be governed and regularly audited [16–18]. This is a difficult and tedious task, as different countries have diverse rules and regulations for sharing and storing medical data [19]. Such differences exist even between hospitals in the same country or departments within a single hospital [20–23]. Centralized append-only or ledger databases, which track changes through cryptographic hashes to prevent tampering of a conventional database, can be used reliably for single hospitals or chains of hospitals to maintain immutable data robustly [24]. These can be further strengthened through digital signatures and trusted timestamping for additional verification [25].

In nephrology, centralized data leads to fragmentation, limiting the vast potential of integrated data such as electronic health records (EHR) and molecular data [26, 27]. Decentralized data handling can improve the integration and linking of datasets. One way to achieve decentralization without using blockchain technology is by copying the data to each institution, partly reducing fragmentation since each institution has access to the same body of data, while keeping the trust centralized to a single institution or administration. For instance, the InterPlanetary File System (IPFS) protocol allows encrypted data to be distributed across multiple authorized data storage nodes, leading to redundancy of the data and for these nodes to be able to verify incoming data [28]. Another way of achieving decentralization is by using distributed ledgers which removed the need for a single central authority, as data-tampering is visible in transaction histories [29]. Blockchain is a specific type of a distributed ledger that organizes these histories in a linear chain of blocks, extending via consensus-driven verification of each block, which is useful when the institutions are mutually distrusting and additions should only be accepted when the institutions agree. An example use-case of consensus-driven verification comes from a study which combined the abstracted findings of fragmented EHR data from multiple hospitals by synchronizing them using blockchain, to implement a system that successfully assists non-nephrologist clinicians to detect undiagnosed CKD across hospitals [30]. Additional studies combined blockchain with machine learning to enable decentralized, multi-center development of models for CKD prediction [31, 32]. These real-world examples show the potential for large-scale data integration in a privacy-preserving manner, with the linearly organized blocks and consensus verification allowing for controlled sequential development of the chain. This contrasts other types of distributed ledgers, where parallel development is approved by a single authority.

Although data integration and decentralization are generally beneficial in collaborative settings, the operational and computational overhead of blockchain integration can be unjustified within an independent hospital or department. In such settings, a well-implemented ledger database with security and interoperability measures (e.g. cryptographic hashing, APIs, and standardized data formatting), can be sufficiently effective.

#### Immutability

The second hallmark of blockchain, immutability, is another crucial part of data integrity and verification. Immutability ensures data, its origin (including how the data was collected and which instruments/procedures were used), and data modifications are verifiable and logged beyond a transaction history. This creates tamper-resistant data storage, increasing trust in the data by all parties involved. However, immutability, by definition, impedes practical data modification, introducing legislative challenges in some regions, such as the ‘right to erasure’ (as is stipulated in Art. 17 of the General Data Protection Regulation (GDPR) of the European Union [23]). This law grants an individual the freedom to withdraw their personal data from a database at any time. Another example of this conflict is when artificial intelligence models are trained using patient data but certain patients withdraw their consent after development of the models [33]. Thus, data amendment and immutability are opposing principles.

**Figure 3:**
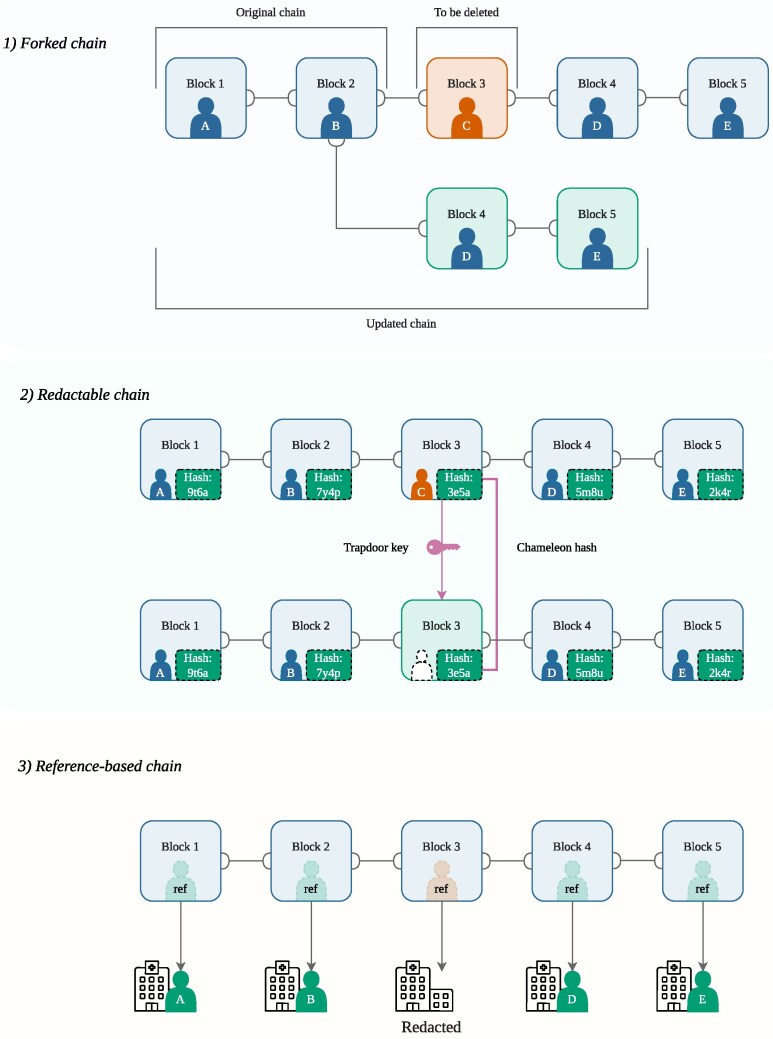
Strategies to address the ‘right to erasure’ in healthcare blockchains.

In real-world scenarios, compromises are often made that both adhere to the GDPR and respect the immutability of a blockchain (Fig. 3) [34]. One option is to ‘fork’ a chain: upon majority consensus, the chain is rebuilt from the block preceding the data to be removed (e.g. data from a particular patient) and recomputing the hashes that came after (now excluding that patient) [35]. Rebuilding the chain is a lengthy, impractical procedure in a healthcare setting, as, for instance, each hospital in the chain might be required to erase patient data when requested. Another technique, called a redactable blockchain, allows an authorized party to use a trapdoor key to generate a ‘chameleon hash’, meaning a redacted data value can replace the original without changing the hash and invalidating the chain [36]. A drawback is that this challenges the decentralized and immutable nature of blockchains and places trust in a single authorized party with a trapdoor key. This becomes a single point of failure for malicious edits if the key is compromised.

A more secure alternative is to keep the data outside the chain and only store the reference to the data on the chain. A real-world example of this is a hash-linked timestamping system used by the Estonian healthcare system since 2016 that is described, although not entirely accurately, as blockchain [28, 37, 38]. Here, a hybrid approach with limited consensus verification is employed: hashes (or references to the data) and timestamps are stored on the chain, while the patient data remain in a modifiable hospital storage system. Since a hash does not contain identifiable information, data erasure is possible without compromising immutability of the hash [28]. This hybrid approach does lead to centralized storage, however, with the possibility of achieving decentralization again through IPFS. The Estonian model relies on a highly coordinated national digital infrastructure, centralized healthcare governance, and long-term governmental investment. Consequently, it should not be interpreted as evidence that similar architectures can be readily implemented in more fragmented healthcare systems or across international nephrology research networks.

Neither extreme of complete centralization nor complete decentralization is a one-size-fits-all solution; an ideal implementation requires legislative compliance and a means to ensure data integrity and integration. The aforementioned workarounds are practical solutions to leverage the security advantages conferred by blockchain while allowing for data modification. Secure and modifiable storage is only the foundation of blockchain in nephrology. More powerful applications emerge through the automation of complex workflows in the governance of big data. In the next section, we explore smart contracts. These represent the next evolution of blockchain technology and enable programmability.

#### Data governance

Large multi-center collaborations accumulating multiple data sources such as EHR, omics and imaging data are becoming more common in nephrology research [27]. While the technical solutions above have addressed the storage, integration, and secure sharing of nephrology data, the management of vast, rapidly-generated quantities of data is a separate challenge. The only feasible way to control data access, data use, and institutional approval processes at scale across clinical networks is to automate the administrative decision-making.

In most current inter-institutional nephrology research settings, data sharing still relies on manual negotiation of data use agreements, institutional review board (IRB) oversight, and coordination across participating sites [39, 40]. These procedures protect patient privacy, but they also delay the progress of collaborative research. In the CKD-EPI clinical trial consortium, one data-sharing agreement took 2 years from the initial verbal agreement to signed agreement, with data transfer only after an additional 12 months [41]. Federated learning faces similar governance limitations. It enables collaborative model training across distributed datasets without sharing patient data, yet requires data governance mechanisms, formal guidelines and agreements to regulate participation [42, 43].

During the initiation phase of research projects or clinical trials in nephrology, as in other areas, researchers often encounter inefficient patient screening, time-consuming ethical approval processes, and limited transparency in data governance [39, 44, 45]. Although some of these processes require clinician oversight, screening eligible participants and, once consent is given, tracking changes in approval or transferring this metadata between systems can be automated. Moreover, in many randomized controlled studies, data must be erased after a retention period [46], which is typically performed manually by researchers. These governance burdens are consistent across the entire research cycle, from patient recruitment to data access and data management after study completion.

Automated screening with predefined criteria and consent tracking is currently possible in many EHR software suites, which significantly increases efficiency over manual screening, reducing cost and time required for manual audits [47]. These can identify eligible patients from within the EHR but typically remain in a single software system of a single institution. Furthermore, changes in consent often need to be synced between different systems and institutions, for example when a hospital develops a machine learning model using data from another hospital’s patient who withdrew their consent. Traditional practices that rely heavily on manual data entry or simple automation are prone to delays and data omissions when moving from one computer system into another [48].

Smart contracts are programmable protocols that automatically execute a predetermined action on the blockchain upon the fulfillment of predefined criteria [49–51]. In one system, smart contract–based matching screened 6 000 patients against predefined criteria, identifying 1 145 eligible participants in under 2 seconds [52]. Using this eligibility screening securely across multiple institutions allows for more efficient cross-institutional recruitment. Additionally, smart contracts can automatically record key research events, such as protocol amendments, data uploads, and audit actions, creating a complete traceable chain of events [50, 51, 53]. In a recently published study, blockchain-based machine learning models were combined to enable multiple hospitals to collaboratively train chronic kidney disease (CKD) detection models. Here, smart contracts were used to manage data access and automatically record every patient data transaction [31]. In kidney transplantation, smart contracts can be programmed to automatically execute cross-matching algorithms after donor and recipient data is uploaded, thereby minimizing manual intervention and reducing the risk of errors [54]. This is demonstrated by the blockchain-enabled organ matching system (BOMS) [55]. This system uses smart contracts to automatically check compatibility between donor and recipient organs, while recording the entire decision-making process on the blockchain. Furthermore, BOMS provides verifiable logs of applied allocation criteria, enhancing trust among patients, donors and regulators [55, 56].

Despite its promising role as an automation tool for governance decisions in nephrology, smart contracts may not be suitable in every context, and their use should be driven by the specific needs of the clinical setting [57]. Within a single institution managed by trusted administrators, data governance can be conducted through conventional institutional data access procedures, local review workflows, and centralized oversight mechanisms [58, 59]. In clinical networks involving multiple institutions, smart contracts may be more valuable because shared access rules and approval conditions need to be implemented across multiple stakeholders and no single party can be given complete control. In this case, distributed ledgers can provide a shared and tamper-resistant record of governance decisions [60]. On this basis, smart contracts can encode governance rules as machine-readable conditions and automatically enforce them during data sharing.

Smart contracts can also support governance in informed consent management, forcing more rigid adherence to legislation. Patients can update the scope, time and purpose of their data usage at any time. Smart contracts automatically verify these changes, perform corresponding access controls, and record all operations in the blockchain to form a complete audit trajectory [61]. In this model, smart contracts not only record the patient’s initial authorization, but they also reflect the permission changes in real time, ensuring that downstream data usage is also immediately compliant with a change in consent [62, 63]. To this end, a recently developed chronic disease management platform uses Hyperledger Fabric technology, enabling configurable authorization templates that are embedded in the platform, so that doctors or researchers can only access data covered by the patient authorization [64]. When permissions change, the system immediately executes and records operations to enable both flexibility and security. One implementation proposes to use smart contracts to combine patient files into Non-Fungible Tokens (NFTs), which function as unique digital assets on the blockchain that cannot be copied or divided. This allows patients to independently give permission for e.g. AI systems to use their data [65, 66].

In another proof-of-concept study, smart contracts have been integrated with mobile applications and IoT sensors to support personalized interventions that trigger when set thresholds in sensing data are exceeded. This can automatically authorize the clinical team to access this abnormal data and initiate instant intervention notifications, such as encouraging the patient to seek medical treatment [67].

Together, these cases demonstrate how smart contracts can encode access rules, automate governance decisions, and make decision workflows auditable in nephrology networks. However, these systems remain at the prototype stage and have not been implemented as routine clinical tools. Taken together, the available evidence suggests that the strongest current rationale for blockchain in nephrology lies in multi-institutional data governance rather than routine clinical care. While pilot studies demonstrate potential benefits for consent management, auditability, and distributed collaboration, evidence that blockchain improves clinical outcomes, healthcare efficiency, or research quality in real-world nephrology practice remains limited.

### Blockchain beyond nephrology data management

**Figure 4:**
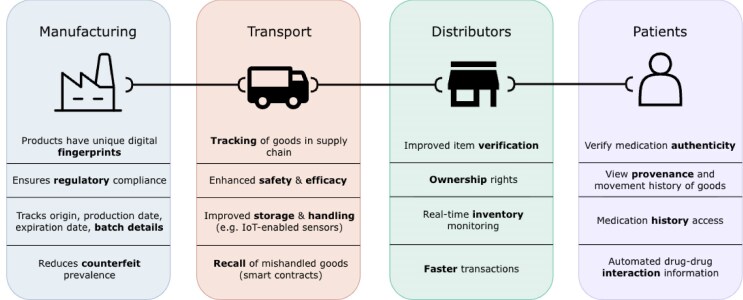
Advantages of end-to-end healthcare supply chain management using blockchains.

Quality assurance of medication and equipment is essential due to the potentially life-threatening consequences of counterfeit or expired drugs and mishandled medical goods in the supply chain, to which nephrological drugs have historically been especially vulnerable [68]. To this end, blockchain infrastructure is being explored as a platform for improved monitoring, tracing, and provenance of medical goods throughout the healthcare supply chain. Pilot projects such as MediLedger, PharmaLedger, and SkyCell have demonstrated the feasibility of blockchain in healthcare supply chain management, including faster pharmaceutical transactions, shorter drug verification times, and improved drug tracing to reduce the risk of counterfeit drugs entering the supply chain [69]. SkyCell has also implemented IoT-enabled refrigeration units that continuously and immutably log temperature, humidity, and geolocation data onto the blockchain during pharmaceutical transport [70]. While promising, the integration of end-to-end IoT-enabled monitoring and transparency to all stakeholders across the healthcare supply chain has yet to be realized in real-world applications, limiting clinical oversight. This represents an important future step towards exploring the full potential of blockchain technology in nephrology. An example of end-to-end blockchain-powered supply chain management is presented in Fig. 4. Beyond supply chain management, blockchain and smart contracts may also benefit nephrology through ensuring reliability of device maintenance records and biobanking data provenance. Nephrology specifically relies on complex medical devices, such as dialysis machines, and imaging systems where blockchain has been shown to mitigate data fragmentation and tampering, while immutably recording to the ledger every inspection, calibration, repair, and software update to optimize the safety, performance, and reproducibility of the devices [71]. Another promising avenue for blockchain in nephrology is for the improved management of biobanks. These repositories of biological patient samples are central to nephrological studies in disease mechanisms, biomarkers, and pharmacological responses. Several studies have highlighted the potential of blockchain to address key limitations of traditional biobanking, including missing samples, incomplete records of sample use or provenance, and dynamic consent management. Blockchain may also support a decentralized network of biobanks, preventing biobank data siloing. However, as in other areas of nephrology, proof-of-concept work is still rarely translated into real-world adoption.

## LIMITATIONS

As discussed earlier, the core benefit of blockchain is to reduce reliance on central authorities. This feature creates challenges for governance in regulated healthcare, where data governance depends on identifiable participants to enable audits, assign accountability, and meet legal obligations [72]. Federated blockchain architectures partially address this challenge by restricting participation to known institutions and defining consensus rules within the network [73]. Yet, such a design challenges the nature of decentralization. Governance in federated clinical networks depends on who controls network access, consensus configuration, smart contract deployment, and policy updates. Therefore, these systems should be viewed as controlled distributed systems, not as fully decentralized ones. Their application in nephrology data governance should be based on a clear assessment of trust, accountability, and control, rather than on the assumption that federated blockchains automatically provide fully decentralized networks. Privacy and regulatory compliance also present challenges. The inherent immutability of blockchain conflicts with the ‘right to erasure’ required by GDPR. Hybrid models that combine off-chain storage with on-chain indexing can partly mitigate this challenge [74]. In these models, deleting off-chain data breaks the on-chain link without modifying the ledger itself. However, this approach introduces new risks. The integrity guarantees provided by on-chain records rely on the security of off-chain storage, which still faces the same centralization and vulnerability issues as any conventional system. For nephrology networks operating across multiple jurisdictions, these uncertainties create barriers to real-world deployment. The implementation of blockchains in real-world clinical research networks requires explicit rules on what information may be stored on-chain. Scalability remains a major concern. Public blockchains such as Ethereum bear long transaction latency and high consumption of computing power and electricity for managing big clinical data [75]. Even permissioned blockchains may face bottlenecks in throughput as the number of nodes rapidly increases [57]. To reduce delay and resource consumption these challenges may cause, developers are testing the use of sharding or layer 2 channels, so that most operations occur off the main chain and the core ledger only records the final summaries [76]. However, these approaches add complexity to the system architecture and remain unvalidated in actual clinical settings for nephrology. In addition, the organizational and economic costs of blockchain deployment also need to be considered. Moving current hospital data infrastructure onto blockchain systems involve software integration, personnel training, ongoing system maintenance, and alignment with existing clinical information systems [77, 78]. These costs need to be balanced against the potential added value of blockchain compared with well-established non-blockchain systems. Importantly, several benefits commonly attributed to blockchain, including data integrity, auditability, and interoperability, can also be achieved through conventional databases, cryptographic verification methods, and distributed data infrastructures without implementing a blockchain. The benefits of blockchain are highly dependent on the broader network in which it is deployed. Distributed ledgers are most useful when participating hospitals and research centers align their technical standards, access policies, and operational workflows. Differences in institutional infrastructure, procurement cycles, and technical expertise may make coordinated deployment slow and costly. This challenge persists even in regions where the adoption of blockchain technology is actively encouraged. In China, despite policy support for blockchain use in healthcare and the release of national clinical guidelines for medical blockchain in 2024, implementation remains largely restricted to pilot projects, without standardized deployment across institutions [78]. Therefore, the use of blockchain in nephrology networks should be guided not only by technical feasibility, but also by structured pilot studies and objective comparisons with simpler alternative solutions, and thorough cost-to-benefit analyses.

## CONCLUSIONS

Blockchain is an emerging technology with potential applications in nephrology, particularly in multi-institutional data governance, auditability, and consent management. However, current evidence is largely limited to pilot studies and proof-of-concept implementations. In some settings, conventional databases, APIs, and established governance frameworks provide comparable functionality with lower barriers for implementation. Future adoption in nephrology should therefore be guided by detailed comparisons with existing solutions, real-world implementation studies, and cost-benefit analyses.

## Data Availability

No new data were generated or analysed in support of this research.
